# 基于集成化多柱二维液相色谱系统分析血清中的氨磺必利

**DOI:** 10.3724/SP.J.1123.2020.07035

**Published:** 2021-02-08

**Authors:** Fenglin WANG, Sandong YANG, Xinying ZHOU, Jiao FENG, Tao TANG, Tong LI

**Affiliations:** 1.大连依利特分析仪器有限公司, 辽宁 大连 116023; 1. Dalian Elite Analytical Instruments Co., Ltd., Dalian 116023, China; 2.中国科学院苏州生物医学工程技术研究所, 江苏 苏州 215163; 2. Suzhou Institute of Biomedical Engineering and Technology, Chinese Academy of Sciences, Suzhou 215163, China

**Keywords:** 多柱二维液相色谱, 氨磺必利, 血清, 治疗药物监测, multi-column two-dimensional liquid chromatography, amisulpride, serum, therapeutic drug monitoring

## Abstract

快速准确的治疗药物监测对于临床上确保患者用药有效性及安全性至关重要,同时也能够确定患者用药依从性,制定个性化给药方案。该文以两支疏水性略有差异的反相分离柱Supersil ODS2和SinoChrom ODS-BP,及强阳离子交换捕集柱Supersil SCX构建了基于集成化的多柱二维液相色谱系统。通过二维色谱接口,以pH 3.0的磷酸缓冲液调整第一维分离后的洗脱液组成,降低有机相含量并维持pH,改善了中心切割模式下样品转移和捕集的效率。利用该多柱二维液相色谱系统发展了血清中氨磺必利的二维液相色谱检测方法,血清样品经过高氯酸和甲醇混合液沉淀蛋白质并离心后直接300 μL大体积进样,以乙腈/磷酸缓冲液(25 mmol/L, pH 3.0)(20/80, v/v)作为第一维分离流动相,磷酸缓冲液(25 mmol/L, pH 3.0)作为捕集过程的稀释流动相,乙腈/磷酸缓冲液(25 mmol/L, pH 7.0)(25/75, v/v)作为第二维分离流动相,12 min内即可完成分析。方法在10~200 ng/mL的范围内线性相关性良好(*r*=0.9998)。样品在50 ng/mL和100 ng/mL两个加标浓度下的回收率稳定,在73.7%~76.8%之间。方法的检出限为7.28 ng/mL,定量限为24.27 ng/mL,能够满足《神经精神药理学治疗药物检测共识指南》中推荐的药物监控范围要求。由于该系统日常使用及维护成本较低,且能够实现自动化分析,故该方法适合在临床上用于治疗药物监测研究。

## Abstract (en)

Amisulpride is a clinically effective antipsychotic drug. It has been recommended for therapeutic drug monitoring in *Consensus Guidelines for Therapeutic Drug Monitoring in Neuropsychopharmacology*. An integrated multicolumn two-dimensional liquid chromatography system was constructed. Two reversed-phase columns, Supersil ODS2 (150 mm×4.6 mm, 5 μm) and SinoChrom ODS-BP (150 mm×4.6 mm, 5 μm), with different hydrophobicities were employed in the first and second separation dimensions, respectively. A strong cation-exchange short column, Supersil SCX (10 mm×4.6 mm, 5 μm) was used to trap samples after the first dimensional separation. A two-position six-port valve was applied to change the flow path for transferring the samples. An auxiliary pump was used to change the mobile phase between the first dimensional column and the trapping column. An intact method for analyzing amisulpride in serum was developed using an integrated multicolumn two-dimensional liquid chromatography system. Serum samples were pretreated only by protein precipitation and centrifugation. In the protein precipitation step, a mixture of perchloric acid (6%, v/v) and methanol was used as the precipitation reagent, whose volume was three times that of the serum sample. The use of this reagent helped eliminate the obvious solvent effects resulting from the large injection volume (300 μL). Then, the samples were vortexed for 2 min and centrifuged for 5 min at a velocity of 10000 r/min. The supernatant was injected into the system directly. Acetonitrile/phosphate buffer (25 mmol/L, pH 3.0; 20∶80, v/v) and acetonitrile/phosphate buffer (25 mmol/L, pH 7.0; 25∶75, v/v) were used as mobile phases for the first and second dimensions, respectively, at a flow rate of 1 mL/min. The solvent strength and pH of the first dimensional eluent were adjusted at the two-dimensional chromatographic interface. Phosphate buffer (25 mmol/L, pH 3.0) was supplied at a rate of 1 mL/min by the auxiliary pump for adjustment. The adjustment process allowed amisulpride to remain cationic, thus leading to improved transfer and trapping efficiencies in strong cation-exchange columns, in the heart-cutting mode. The trapping time was determined to be between 4 and 5 min by a confirmation experiment. The use of a short trapping column and the appropriate mobile phase conditions allowed us to complete the analysis within 12 min. The established method was validated in detail by investigating the linearity, limit of detection (LOD), limit of quantification (LOQ), and recovery. A good linear relationship was observed between 10 and 200 ng/mL (*r*=0.9998). The LOD and LOQ were 7.28 ng/mL and 24.27 ng/mL, respectively. The high sensitivity of the validated method met the requirements of the therapeutic reference range of the *Consensus Guidelines for Therapeutic Drug Monitoring in Neuropsychopharmacology*. The recovery of amisulpride spiked in a serum sample at 50 and 100 ng/mL were stabilized between 73.7% and 76.8%, which revealed the good stability of the established method. As opposed to the complicated traditional analytical methods, our method based on the automated integrated system is cost-effective and low-maintenance, thus being appropriate for routine therapeutic drug monitoring in clinical research. Moreover, our method is highly promising as the system cost is much lower than that of the popular LC-MS, while the capacity is sufficient for the determination of drugs in serum.

氨磺必利是一种苯酰胺类第2代抗精神病药物,可选择性地与多巴胺D2、D3受体结合,通过阻断多巴胺D2、D3受体,改善精神分裂症阴性及阳性症状^[1]^。德国神经精神药理学与药物精神病学协会^[2]^(Arbeitsgemeinschaft für Neuropsychopharmakologie und Pharmakopsychiatrie, AGNP)在2017年更新的《神经精神药理学治疗药物检测共识指南》(以下简称《指南》)中推荐的血液中有效治疗浓度范围是100~320 ng/mL,并将其治疗药物监测(therapeutic drug monitoring, TDM)推荐等级列为一级。由于血液基质比较复杂且药物浓度较低,为了减小其他物质对血液中药物浓度检测的干扰,提高检测灵敏度,多采用不同的前处理技术结合色谱法及高灵敏度检测器进行分析检测^[3,4]^。陈颖等^[5,6]^分别以乙醚和乙酸乙酯萃取血浆与血清中的氨磺必利等治疗药物,由于萃取溶剂用量较大,需要采取氮吹、复溶等额外操作控制进样量及检测灵敏度,血清基质下的定量限达到了25 ng/mL。Kudris等^[7]^发展了一种离线固相萃取结合液相色谱-荧光检测器分析血浆中氨磺必利的方法,利用固相萃取柱对血浆样品进行离线净化富集,净化富集后的样品经过蒸干、复溶后进样分析,以高灵敏度的荧光检测器进行检测,定量限可达10 ng/mL。液相色谱-质谱联用技术由于具有较高分辨率和灵敏度,也常用于治疗药物的监测^[8,9]^。Mokhtar等^[10]^利用超高效液相色谱-离子肼质谱同时检测血液中113种药物的浓度,其中氨磺必利的检出限为3 ng/mL,但样品处理过程同样需要液液萃取、氮吹、复溶等操作。这些具有复杂前处理过程的分析方法尽管能够满足《指南》要求的检测浓度范围,但前处理过程繁琐耗时,分析效率低,且处理过程中容易产生样品的损失,不利于方法的推广。此外,质谱检测虽然具有极高的检测灵敏度,但较高的仪器价格却会限制其应用扩展,同时,质谱定量常用的同位素内标试剂也增加了日常检测的成本^[11]^。

多柱二维液相色谱是分离分析复杂样品的重要工具^[12]^,在蛋白质组学^[13]^、代谢组学^[14]^、中草药^[15]^分析等领域有广泛的应用,同样也适用于在复杂的血液基质中分析药物组分^[16]^。由于二维液相色谱是多种分离模式的组合,因此比常规一维色谱具有更好的分离能力,能够实现待测物与干扰组分的快速分离。此外,二维液相色谱也可以基于切换阀结构实现大体积样品的在线富集、中心切割样品转移以及自动化分析,可减少人为因素的干扰,具有更高的灵敏度和分析效率^[17]^。尚翔等^[18]^利用二维液相色谱建立氨磺必利血药浓度检测方法,并对患者的血药浓度、服药剂量、年龄、性别之间的关系进行了分析。

本文采用中心切割的样品转移接口形式,构建了集成化的多柱二维液相色谱系统,兼具大体积样品直接进样、辅助泵稀释多维样品转移以及全模块自动化控制等功能,并基于该系统对血清中氨磺必利的检测进行了方法学研究。

## 1 实验部分

### 1.1 仪器、试剂与材料

3台P1100高压恒流泵,D1100紫外-可见检测器,S3100自动进样器,W5100色谱工作站,色谱柱均为大连依利特分析仪器有限公司产品。两位六通切换阀(C82X-6676, VICI)。高速离心机(3K15, Sigma)。紫外分光光度计(UV2900,上海舜宇恒平)。

乙腈(色谱纯,Sigma),纯化水(Milli-Q),磷酸二氢钾、磷酸氢二钾(分析纯,科密欧),高氯酸(分析纯,70%,科密欧),氨磺必利对照品(>98%,阿拉丁)。血清样品使用前储存在-20 ℃冰箱中。

### 1.2 标准溶液配制及样品前处理

标准储备液(1 mg/mL):称取10.0 mg氨磺必利对照品于10 mL容量瓶中,甲醇溶解并定容。

标准工作液:取标准储备液适量,用6%(v/v)高氯酸逐级稀释成质量浓度为10、25、50、100、200 ng/mL的标准溶液。

血清样品前处理:将血清样品从冰箱中取出,自然解冻。取血清样品1 mL,加入3倍体积的高氯酸(6%, v/v)/甲醇(85/15, v/v)^[19]^,涡旋2 min, 10000 r/min高速离心5 min,取上清液进样分析。

### 1.3 色谱条件

第一维分析柱,Supersil ODS2(150 mm×4.6 mm, 5 μm);第二维分析柱,SinoChrom ODS-BP(150 mm×4.6 mm, 5 μm);捕集柱,Supersil SCX(10 mm×4.6 mm, 5 μm)。进样量:300 μL;检测波长:280 nm。

流动相A,乙腈/磷酸缓冲液(25 mmol/L, pH 3.0)(20/80, v/v), 1.0 mL/min。流动相B,磷酸缓冲液(25 mmol/L, pH 3.0),流速梯度:0~4 min, 0.1 mL/min; 4~5 min, 1.0 mL/min; 5~12 min, 0.1 mL/min。流动相C,乙腈/磷酸缓冲液(25 mmol/L, pH 7.0)(25/75, v/v), 1.0 mL/min。

切换阀0~4 min,实线位;4~5 min,虚线位;5~12 min,实线位(见图1)。

**图1:**
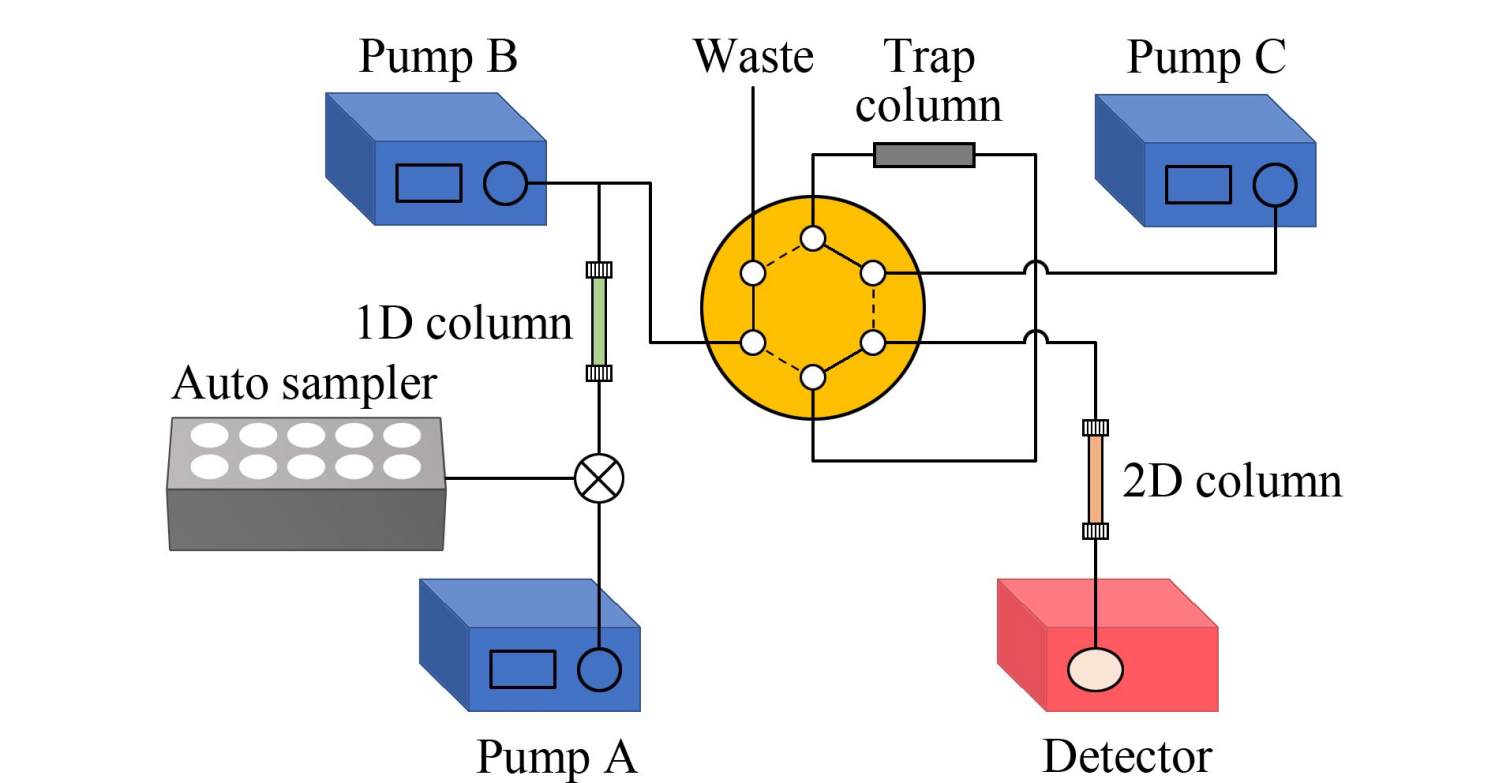
多柱二维液相色谱系统结构示意图

## 2 结果与讨论

### 2.1 集成化多柱二维液相色谱系统的构建

系统结构如图1所示。输液泵A、B、C分别输送流动相A、B、C。初始条件下,切换阀处于实线位,优先从第一维色谱柱上洗脱下的非待测组分通过切换阀排入废液流路。当待测组分被洗脱下时,切换阀切换至虚线位,使第一维色谱柱出口与捕集柱相通。同时,通过三通与第一维色谱柱出口相连的输液泵B流量提高,稀释第一维洗脱下的溶剂强度。这种接口结构能够使待测组分更多地保留在捕集柱上,提高样品转移与捕集的效率,避免有机相比例过高引起样品的展宽和损失。捕集结束后,切换阀切换回实线位,由输液泵C将捕集柱上的待测组分洗脱至第二维色谱柱上进行进一步分离。由于实验所用的色谱工作站能够同时控制系统中的所有模块,并且可以序列运行,因此该集成化多柱二维液相色谱系统能够实现自动化分析。

### 2.2 氨磺必利紫外吸收光谱测试

利用紫外可见分光光度计对氨磺必利进行光谱图扫描,结果发现在紫外区有2个特征吸收波长,分别为226 nm和280 nm。其中226 nm响应值相对较高,但考虑到此区域干扰较大,因此选择次大吸收峰位置280 nm作为检测波长。

### 2.3 氨磺必利标准品的一维分析结果

以1 μg/mL氨磺必利标准品进样,考察不同进样体积对保留时间峰面积的影响,叠加谱图见图2。随着进样体积的增大,标准品色谱峰的保留时间逐渐增加,但峰面积与进样量的线性关系依然良好(*r*=0.997)。保留时间的延长可能与样品溶剂的影响有关,随着进样体积的增大,样品溶剂(6%(v/v)高氯酸)会逐渐降低流动相的洗脱强度,使样品保留加强。但由于进样体积的增大并未造成明显的溶剂效应,使峰形变差,因此可以通过增加进样量的方式提高检测灵敏度。

**图2:**
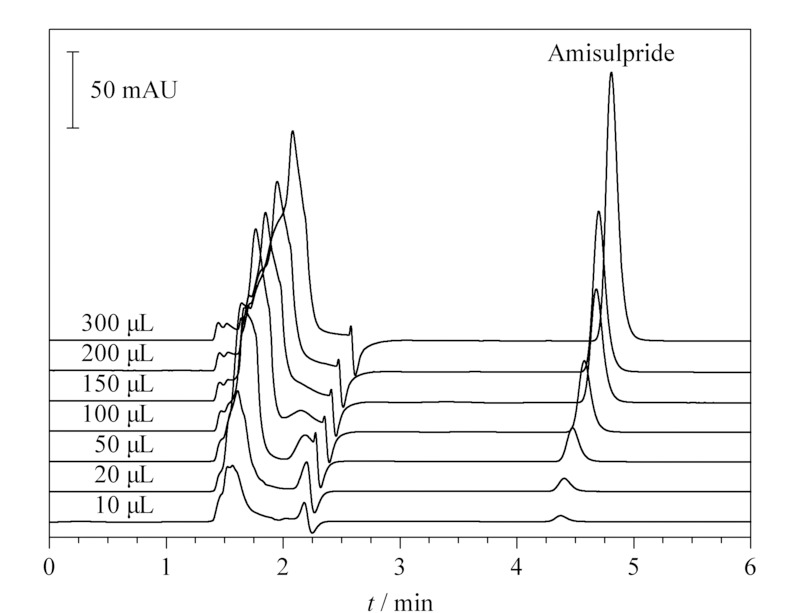
不同进样体积的1 μg/mL氨磺必利标准溶液的色谱图

以接近检出限要求^[2]^的100 ng/mL标准液为分析对象,进一步考察直接进样及增加捕集柱和输液泵B后出峰时间的差异性,以便准确设定捕集柱切入系统时间,结果见图3。单独进样时,标准品保留时间为4.53 min,半峰宽为0.11 min(峰起落点4.32~4.82 min)。增加捕集柱及开启输液泵B后,保留时间为5.84 min,半峰宽0.31 min(峰起落点5.44~6.18 min)。因此,基于上述数据,设定切入捕集柱时间范围为4.00~5.00 min,可以有效保证目标样品均能完成捕集,并且不会从捕集柱上二次洗脱下来。

**图3:**
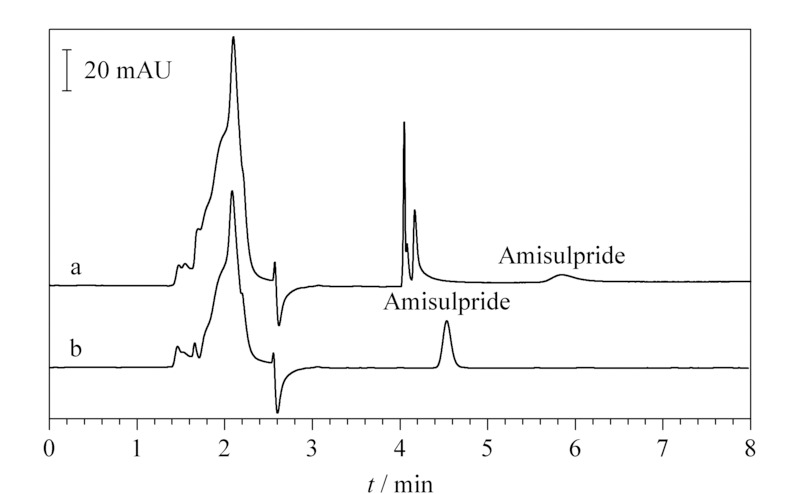
100 ng/mL氨磺必利标准溶液在(a)接捕集柱与(b)不接捕集柱时的色谱图

### 2.4 多柱二维系统分析氨磺必利

2.4.1 二维分析方法

按照1.3节所述方法,系统的第一维需要对样品进行快速拆分,因而使用疏水性较弱的Supersil ODS2反相色谱柱。以pH 3.0的磷酸缓冲液和乙腈混合作为第一维流动相,可使氨磺必利带正电,进一步降低其在第一维反相柱上的保留,同时实现了对加标血清样品的初步分离。通过中心切割的方式,可将带正电的氨磺必利与极性相近的组分转移至SCX捕集柱上。为了降低第一维洗脱液中乙腈对样品捕集的影响,以pH 3.0的流动相B对洗脱溶液进行稀释,同时维持体系的pH,确保带正电的氨磺必利能够在SCX捕集柱上保留。捕集结束后,样品被转移至第二维进行进一步分离。第二维所用的色谱柱填料为SinoChrom ODS-BP,疏水性较强,并且在C18键合填料的基础上使用了碱性封尾技术,降低硅羟基残留,更加适合碱性物质的分离,改善峰形。为了加快分析速度,SCX捕集柱使用了10 mm的短柱;同时,第二维流动相使用pH 7.0的磷酸缓冲液和乙腈的混合液,不仅可降低氨磺必利的解离程度,使其能够在SCX捕集柱上被快速地洗脱下来,还能够改善氨磺必利在第二维反相柱上的保留行为,实现其与杂质的基线分离。

2.4.2 标准液的二维分析结果

按照1.3节二维系统流程和色谱条件,对10、25、50、100、200 ng/mL的氨磺必利标准液进行了分析,以峰面积(*y*)对质量浓度(*x*, ng/mL)进行线性回归,得线性方程为*y=*1.54*x*-0.91,*r*为0.9998,相关性良好。

2.4.3 加标血样的分析结果

加标血清样品的叠加谱图如图4所示。在50 ng/mL和100 ng/mL两个加标水平下,3个平行试验的回收率在73.7%~76.8%之间,回收率虽然不是特别高,但结果稳定(RSD不大于1.6%)。样品采集基线噪声实测为0.05 mAU,根据50 ng/mL加标测试平均峰高(1.03 mAU)测算,方法检出限(*S/N*=3)为7.28 ng/mL,定量限(*S/N*=10)为24.27 ng/mL。

**图4:**
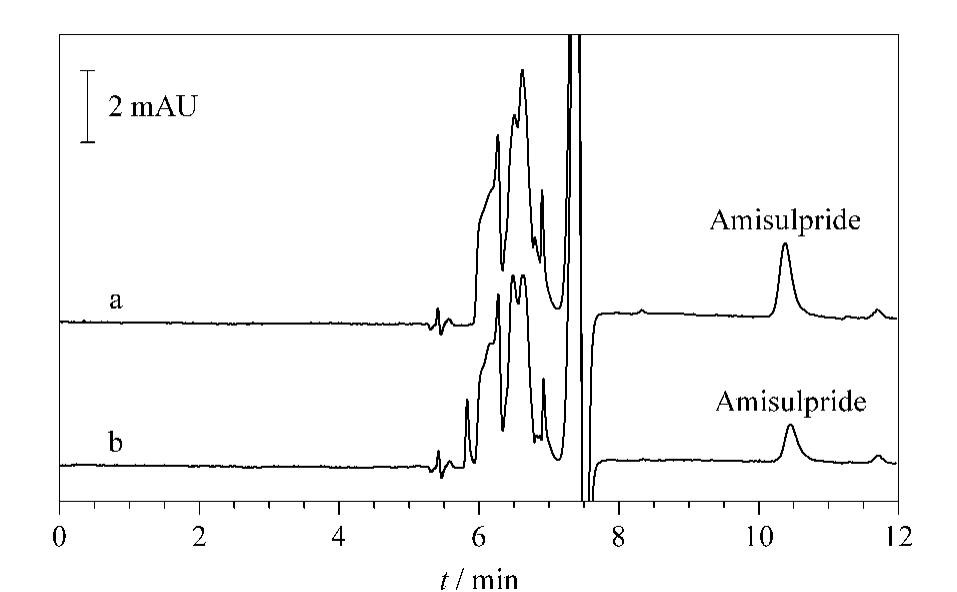
不同水平加标血样分析的色谱图

## 3 结论

本文以中心切割模式构建了集成化多柱二维液相色谱系统,同时在样品接口处增加旁路,以辅助泵调节第一维洗脱下的样品溶剂强度,使样品能够有效地捕集在捕集柱上。以二维模式分析血清中的氨磺必利,12 min内可以完成样品分析。氨磺必利在10~200 ng/mL范围内线性相关性良好,加标回收率稳定,定量限为24.27 ng/mL,满足100~320 ng/mL的AGNP推荐药物监控范围要求(低于监控范围的下限)。所构建的血药浓度多柱二维分析系统能够在较短的时间内在线去除血清中大部分的基质干扰,减少了血清样品的前处理流程,确保待测组分的分离度。同时仪器价格、使用及维护成本均远低于液相色谱-质谱联用系统,适合在临床血药浓度监测领域推广使用。
